# Transspinal temporal interference stimulation enhances neuroplasticity-associated responses and facilitates motor recovery after spinal cord injury in mice

**DOI:** 10.3389/fnins.2026.1950023

**Published:** 2026-10-06

**Authors:** Peng Zheng, Yan Chen, Yujiao Yue, Xianbin Wang, Shuang Wu

**Affiliations:** 1 Clinical Medical College, Guizhou Medical University, Guiyang, Guizhou, China; 2 Department of Rehabilitation Medicine, Affiliated Hospital of Guizhou Medical University, Guiyang, Guizhou, China; 3 Department of Rehabilitation Medicine, Affiliated Hospital of Zunyi Medical University, Zunyi, Guizhou, China

**Keywords:** axonal growth, neuromodulation, neuroplasticity, spinal cord injury, synaptic plasticity, temporal interference stimulation

## Abstract

**Introduction:**

Spinal cord injury (SCI) causes substantial motor impairment, driven by neuronal loss, axonal disruption, and impaired synaptic connectivity. Temporal interference (TI) stimulation is capable of modulating neural activity, but its therapeutic potential in SCI remains largely unexplored. We explored the influence of TI stimulation on functional recovery and neuroplasticity-associated responses following SCI.

**Methods:**

Thirty-two C57BL/6J mice were randomly allocated to four groups: sham, SCI, sham stimulation, and TI stimulation. Mice underwent T8 spinal cord hemisection, followed by daily TI stimulation from day 4 to day 21 post-injury. Locomotor recovery was evaluated using the Basso Mouse Scale (BMS). At 21 days after injury, neuronal-associated changes, glial responses, and neuroplasticity-associated molecular changes were assessed by immunofluorescence staining and western blot analysis.

**Results:**

Compared with sham-operated mice, SCI mice exhibited impaired locomotor function, reduced NeuN-positive neuronal density, and decreased expression of neuroplasticity-associated proteins, including GAP43, NF-L, SYN, and PSD95. In contrast, TI stimulation significantly improved BMS scores and increased NeuN-positive neuronal density, accompanied by enhanced expression of axonal growth-associated proteins (GAP43 and NF-L) and synaptic plasticity-associated markers (SYN and PSD95).

**Discussion:**

These findings indicate that TI stimulation improves functional recovery after SCI and is associated with enhanced neuroplasticity-related responses, including axonal growth-associated and synaptic plasticity-associated changes. This study provides evidence supporting TI stimulation as a promising neuromodulation strategy for promoting neuroplasticity-associated recovery processes after spinal cord injury.

## Introduction

Spinal cord injury (SCI) represents a major neurological challenge, often causes persistent long-lasting functional impairments (McDonald and Sadowsky, 2002; Müller-Jensen et al., 2021). Severe SCI can lead to lifelong disability and represents a substantial socioeconomic burden worldwide (Jiang et al., 2021; Lu et al., 2025). Following SCI, complex pathological cascades occur at the injury site, involving both primary and secondary injury processes (McDonald and Sadowsky, 2002; Hu et al., 2023). Primary injury is characterized by immediate direct damage, including neuronal loss, vascular disruption, axonal transection, and disruption of synaptic connectivity (Alizadeh et al., 2019; Anjum et al., 2020). Subsequently, secondary injury processes, including neuroinflammation, demyelination, ischemic responses, and oxidative stress, further amplify tissue damage and create an inhibitory microenvironment that restricts axonal remodeling and synaptic plasticity, thereby limiting neurological recovery (Ahuja et al., 2017; Orr and Gensel, 2018; Hellenbrand et al., 2021). Animal models are essential tools in biomedical research, providing important platforms for investigating disease mechanisms and evaluating potential therapeutic strategies before clinical translation (Choudhary, 2025; Choudhary and Sarkar, 2025).

Axonal regeneration and synaptic remodeling are essential processes underlying neurological recovery after SCI, as they contribute to the restoration of disrupted neural connectivity and the reconstruction of functional circuits (Hu et al., 2023). However, unlike peripheral nerves, injured axons in the developed nervous system display poor regenerative capacity due to multiple intrinsic and extrinsic constraints. These limitations include the failure to establish a permissive growth environment across the lesion site, the presence of inhibitory molecules within myelin and extracellular matrix, reactive glial responses, and insufficient activation of intrinsic neuronal growth programs (O'Donovan, 2016; Zheng and Tuszynski, 2023). In parallel with axonal disruption, SCI causes extensive loss of synaptic connections and impairs synapse formation, maturation, and adaptive plasticity, thereby limiting efficient neural transmission and functional recovery (Anderson et al., 2018; Wu et al., 2025). Increasing evidence indicates that neural plasticity, including axonal sprouting and synaptic reorganization, plays a critical role in neural function restoration after SCI, highlighting the therapeutic potential of strategies that promote adaptive circuit remodeling (Harkema, 2001; Behrman et al., 2017; Wagner et al., 2018; Anderson et al., 2022).

Given the intrinsic capacity of the injured nervous system for adaptive remodeling, in the context of SCI, various therapeutic strategies, including stem cell-based therapies, have been explored to promote neural repair and functional recovery (Mili and Choudhary, 2024). However, challenges such as poor cell survival and insufficient integration with host neural circuits continue to limit the clinical translation of stem cell-based approaches for SCI (Abusalah et al., 2024). Therefore, neuromodulation-based interventions have drawn considerable attention in SCI research due to their potential to modulate neural circuits and enhance neural repair (Pizzolato et al., 2021; Zhang et al., 2021). Existing approaches, such as epidural electrical stimulation (EES), transcutaneous spinal cord stimulation (TSCS), and repetitive transcranial magnetic stimulation (rTMS), have shown potential in improving motor outcomes after SCI by engaging preserved neural circuits and promoting adaptive neural plasticity (Choi et al., 2021; Minassian et al., 2024; Ren et al., 2025; Fu et al., 2025). Non-etheless, these techniques remain constrained by limitations such as invasiveness, insufficient penetration depth, limited spatial selectivity, and challenges in precisely targeting deep spinal neurocircuitry. Temporal interference (TI) stimulation is an emerging neuromodulation strategy that utilizes the interaction of two high-frequency alternating currents to induce a low-frequency envelope-modulated electric field within targeted tissues, enabling spatially selective modulation of targeted neural tissues while potentially reducing unintended activation of surrounding tissues (Grossman et al., 2017). Unlike traditional electrical stimulation approaches, TI stimulation provides an emerging approach for spatially selective modulation of neural activity within targeted neural tissues. Recent studies have suggested that TI stimulation can regulate neuronal excitability and influence neuroplastic processes, potentially through modulation of synaptic protein expression, neurotransmitter release, dendritic spine remodeling, and synaptic vesicle dynamics (Qi et al., 2024; Wang et al., 2025b; Peressotti et al., 2026). These researches imply that TI stimulation may possess the capacity to regulate neural plasticity; however, whether such effects can be translated into structural and molecular changes associated with neural repair after SCI remains unknown. Although TI stimulation has shown promising applications in neurological disorders and has recently been explored in peripheral nerve modulation, its therapeutic potential in SCI remains largely unexplored. Specifically, whether TI stimulation can enhance axonal growth-associated responses and synaptic plasticity-related changes to facilitate functional recovery after SCI remains unclear.

Therefore, this study aimed to explore whether transspinal TI stimulation could promote motor function after SCI and whether these effects were related to changes in NeuN-positive neuronal density and modulation of axonal growth-associated and synaptic plasticity-related responses. We hypothesized that TI stimulation may facilitate neurological recovery after SCI by modulating neuronal, axonal growth-associated, and synaptic plasticity-related responses.

## Materials and methods

### Materials and equipment

Materials: BCA protein assay kit (WB6501, NCM, China), RIPA lysis buffer (R0010, Solarbio, China), Four-color multiplex immunofluorescence staining kit (TSA-based) (AFIHC024, Aifang Biotechnology, China), and 1% sodium pentobarbital (Sinopharm Chemical Reagent Co., Ltd., China). Primary antibodies against β-actin, GAPDH, SYN, and NeuN (20536-1-AP, 10494-1-AP, 17785-1-AP, and 66836-a-lg, Proteintech, China), PSD95 and GAP43 (ET1602-20 and ET1610-94, HUABIO, China), NF-L (EPR22035-112, Abcam, USA), and GFAP (R22776, ZenBio, China).

Equipment: temporal interference stimulation device (NervioX-1000, Xi'an Qiongdong Medical Technology Co., Ltd., China), Cryostat microtome (Epredia, USA), chemiluminescence imaging system (Bio-Rad, USA), electrophoresis system (Bio-Rad, USA), and laser scanning confocal microscope (Olympus, Japan).

### Animals and experimental design

Adult female C57BL/6J mice (6–8 weeks) were supplied from Guizhou Chaoyu Biotechnology Co., Ltd. (Zunyi, China; License No. SYXK (Gui) 2025-0001). The mice were kept under specific conditions (22–26 °C, 40%−60% relative humidity, and a 12-h light/dark cycle) with free access to food and water. Subsequently, animals were allocated into four groups (*n* = 8/group): the sham group, SCI group, SCI + sham stimulation group (SCI+SS), and SCI + TI stimulation group (SCI+TI).

The sample size was determined based on previous studies using similar spinal cord injury models and to account for potential experimental loss during the study period. No animals died during the experimental period, although some spinal cord samples were unsuitable for subsequent analysis due to tissue damage during collection. Behavioral assessments were performed in all animals (n = 8/group). For endpoint tissue analyses, three independent animals per group were used for immunofluorescence staining, and another three independent animals per group were used for Western blot analysis. Tissue sample selection and quantitative analyses were performed by investigators blinded to group allocation. Throughout the manuscript, n represents the number of independent animals rather than the number of sections, microscopic fields, or technical replicates.

Sham animals received surgical exposure only, with no spinal cord lesion created and no subsequent treatment administered. The SCI group underwent SCI induction and received no stimulation treatment. The SCI+SS group and SCI+TI group underwent SCI induction followed by sham stimulation or TI stimulation, respectively. Detailed procedures for TI stimulation and sham stimulation are described in the TI stimulation protocol section. Figure 1 provides a schematic representation of the experimental design.

**Figure 1:**
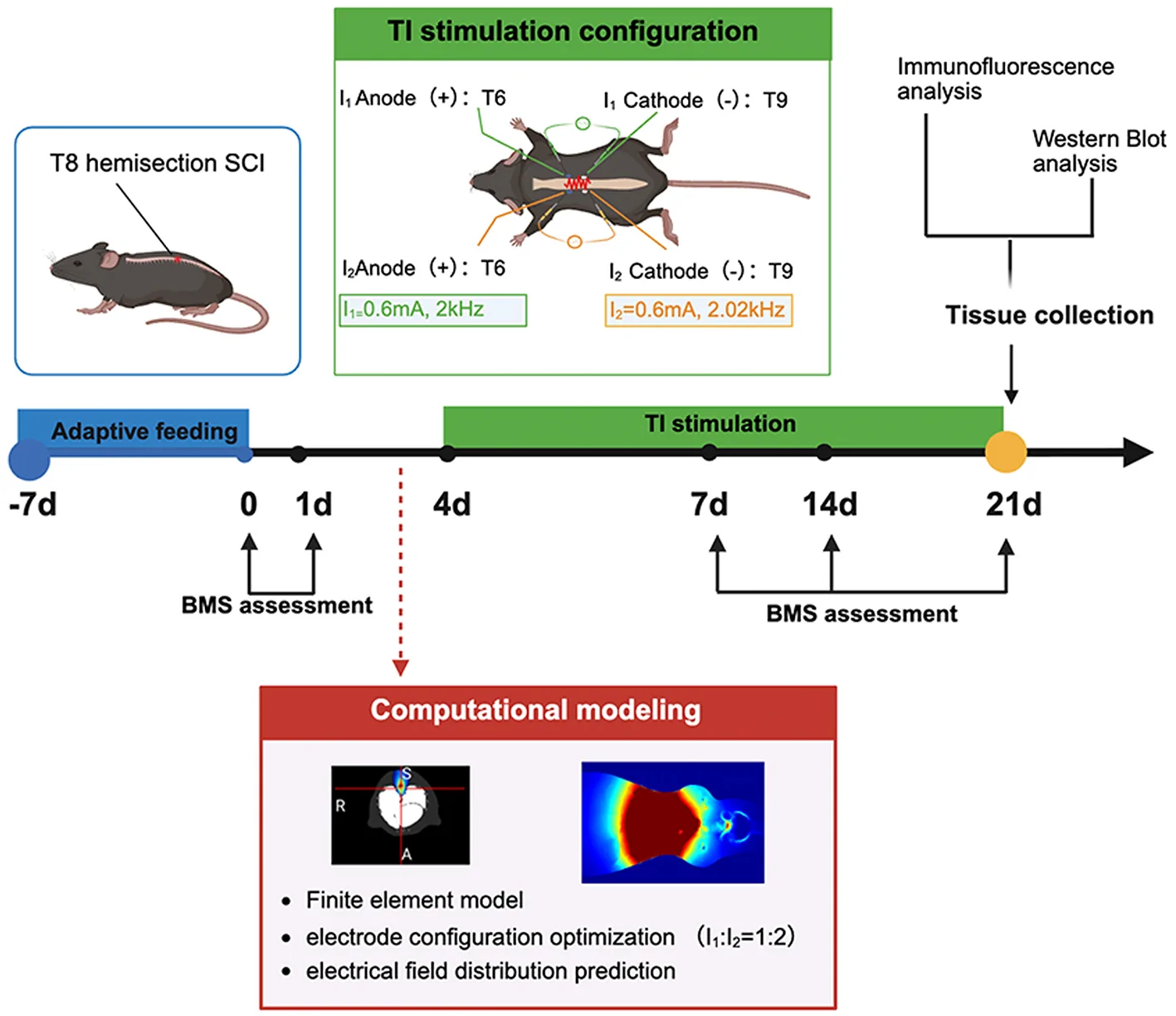
Experimental design and timeline.

### SCI model establishment

Following anesthesia, the dorsal hair over T7–T9 vertebral region was removed from mice. The animals were positioned prone and securely fixed on a surgical platform. Following surgical preparation, the T8 spinal cord was exposed through a dorsal approach after muscle separation and partial laminectomy. The dorsal median sulcus was used as the midline reference. A standardized right lateral hemisection injury was then induced by excising a 1.0 ± 0.3 mm-long portion of the right lateral spinal cord tissue using fine microsurgical scissors.

After surgery, the incision was closed anatomically, and animals received bladder assistance twice daily until spontaneous urination returned. Sham animals received identical exposure surgery without SCI, while the residual interventions and postoperative care were identical to the SCI group.

### TI stimulation protocol

Transspinal stimulation procedures were performed under inhalation anesthesia using insulated needle electrodes positioned at the paraspinal regions of the targeted spinal segments. Mice were initially anesthetized with 2-3% isoflurane for anesthesia induction and subsequently maintained with 1.5–2% isoflurane throughout the stimulation procedure. After anesthesia induction, mice were placed in a prone position on a heating pad to maintain body temperature, and the electrodes were inserted according to the predefined stimulation configuration.

The electrodes were placed at the paraspinal regions of T6 (anode) and T9 (cathode) to target the T6–T9 spinal cord region. The electrode configuration was designed based on computational modeling, and the resulting electric field distribution was validated prior to animal experiments. Detailed information regarding the computational modeling and electric field distribution is presented in Figure S1.

Two alternating currents with frequencies of 2 kHz and 2.02 kHz were applied through the electrodes, with stimulation amplitudes of I1 = 0.6 mA and I_2_ = 0.6 mA, respectively. The frequency difference between the two carrier currents generated a 20 Hz temporal interference envelope within the targeted spinal cord region. Each stimulation session consisted of a 20-s ramp-up phase, continuous stimulation, and a 20-s ramp-down phase, resulting in a total duration of 20 min and 20 s. TI stimulation was initiated on post-injury day 4 and administered once daily until post-injury day 21, resulting in 18 consecutive stimulation sessions.

For sham stimulation, mice in the SCI+SS group underwent the same anesthesia procedures, positioning, electrode placement, and handling procedures as the SCI+TI group. However, no electrical current was delivered during the stimulation sessions. Sham stimulation was performed with the same stimulation duration and experimental schedule as TI stimulation to control for potential effects associated with anesthesia, electrode insertion, and experimental manipulation.

### Locomotor function assessment

Locomotor function restoration of the hindlimbs was evaluated using the Basso Mouse Scale (BMS) before surgery and at 1, 7, 14, and 21 days after injury (dpi). This 10-point ordinal scale ranges from 0, representing complete hindlimb paralysis without voluntary movement, to 9, indicating normal hindlimb locomotion.

### Tissue collection and preparation

Mice were subjected to anesthesia with 0.3% pentobarbital sodium delivered intraperitoneally. According to the requirements of subsequent experiments, spinal cord tissues were collected using either non-perfusion or perfusion procedures.

For Western blot analysis, the vertebral column was rapidly exposed, and a spinal cord segment approximately 1 cm in length, centered on the T8 level (approximately 0.5 cm rostral and 0.5 cm caudal to the lesion), was carefully collected. Following tissue collection, samples were immediately snap-frozen in liquid nitrogen and maintained at −80 °C until subsequent analysis.

For immunofluorescence staining, mice underwent transcardial perfusion through the left ventricle with an incision in the right atrium. The animals were first perfused with physiological saline until the effluent became clear, followed by fixation with 4% paraformaldehyde (PFA). After perfusion, spinal cord tissue encompassing the lesion region were collected and post-fixed in 4% PFA for 24 h. The tissues were subsequently soaked in 20% and 30% sucrose solutions until they sank completely. Then tissues were embedded in optimal cutting temperature compound for subsequent sectioning.

### Immunofluorescence analysis

Frozen spinal cord sections were rinsed with PBS and subsequently subjected to antigen retrieval and permeabilization procedures. After blocking endogenous peroxidase activity with a peroxidase blocking solution, sections were washed with PBS and subjected to multiplex immunofluorescence staining using the TSA method. GFAP/NeuN and PSD95/SYN were examined in separate double-staining sets.

For the first round of staining, sections were incubated overnight at 4 °C with primary antibody against GFAP (1:1000) or PSD95 (1:2000), diluted in 10% goat serum. After washing with PBS, sections were incubated with HRP-conjugated secondary antibodies, followed by TSA fluorophore staining. To remove the bound antibody complexes, sections were incubated in antibody stripping buffer and then washed with PBS. The stripping procedure was repeated once, followed by incubation with peroxidase blocking solution for 15 min to eliminate residual HRP activity.

The second round of staining was performed following the same protocol as the first round, with primary antibody against NeuN (1:1000) or SYN (1:5000). Secondary antibody incubation and TSA amplification were conducted according to the procedures described above. Finally, sections were counterstained with DAPI, mounted with antifade mounting medium, and imaged using a fluorescence microscope.

For quantitative analysis, three representative spinal cord sections containing the lesion epicenter were selected from each animal. A 10 × overview image was first acquired for lesion localization, followed by acquisition of 20 × images from the caudal perilesional region adjacent to the lesion epicenter. The same anatomical criteria were applied for ROI selection and image acquisition across all groups. Image acquisition and quantitative analyses were performed by investigators blinded to group allocation. Although multiple sections and images were analyzed, the individual animal was considered the experimental unit for statistical analysis.

Image quantification was conducted using Fiji (ImageJ) software. GFAP expression was quantified as the percentage of GFAP-positive area in the analyzed region. NeuN-positive cells were quantified as neuronal density, calculated as the number of NeuN-positive cells per unit area. PSD95 and SYN expression levels were quantified based on mean fluorescence intensity. All images were captured using identical acquisition parameters, and quantitative analyses were conducted using consistent threshold settings.

### Western blot analysis

Following homogenization in lysis buffer containing 1% PMSF, tissue lysates were centrifuged at 12,000 rpm for 15 min at 4 °C. The resulting supernatants were collected for protein quantification using the bicinchoninic acid assay.

Protein samples were resolved by SDS-PAGE and electroblotted onto PVDF membranes. After blocking with 5% skim milk for 1 h at room temperature, membranes were incubated with the indicated primary antibodies at 4 °C overnight: β-actin (1:5000), GAPDH (1:5000), GAP43 (1:3000), NF-L (1:1000), PSD95 (1:3000), and SYN (1:5000). After washing, the membranes were incubated with HRP-conjugated secondary antibodies (1:5000) for 1 h at room temperature. Protein bands were visualized using an enhanced chemiluminescence detection system. The band intensities of GAP43, NF-L, PSD95, and SYN were quantified using Fiji software and normalized to the corresponding loading controls.

### Statistical analysis

Statistical processing was carried out in GraphPad Prism 10.0. Data are shown as mean ± standard deviation (SD). Group comparisons were performed using one-way ANOVA followed by Tukey's *post hoc* analysis. Changes in BMS scores over time were assessed by two-way repeated-measures ANOVA with Geisser–Greenhouse correction. When a significant time × group interaction was detected, simple main-effects analyses were performed using one-way ANOVA, followed by Tukey's multiple-comparisons test. Partial eta squared (ηp^2^) was reported as the effect size for ANOVA. A threshold of *P* < 0.05 indicated statistical significance.

## Results

### TI stimulation improves hindlimb locomotor recovery after SCI

Hindlimb locomotor recovery following SCI was assessed using the BMS before surgery and at 1, 7, 14, and 21 dpi to evaluate the therapeutic effects of TI stimulation. Baseline BMS scores were comparable among all groups before surgery.

Two-way repeated-measures ANOVA revealed a significant time × group interaction in BMS scores (*F* (6.497, 60.64) = 21.16, *P* < 0.0001, ηp^2^ = 0.694). Simple main-effects analyses showed significant group effects at 7 dpi (*F* (3, 28) = 981.9, *P* < 0.0001, ηp^2^ = 0.991), 14 dpi (*F* (3, 28) = 880.8, *P* < 0.0001, ηp^2^ = 0.990), and 21 dpi (*F* (3, 28) = 420.7, *P* < 0.0001, ηp^2^ = 0.978).

At 1 dpi, BMS scores were markedly reduced in the SCI, SCI+SS, and SCI+TI groups compared with the sham group. No significant differences were observed between the SCI+TI and SCI or SCI+SS groups at 7 dpi (both adjusted *P* = 0.3197). In contrast, BMS scores were significantly higher in the SCI+TI group than in the SCI and SCI+SS groups at both 14 dpi (adjusted *P* = 0.0005 and 0.0066, respectively) and 21 dpi (adjusted *P* = 0.0048 and 0.0051, respectively) (Figure 2).

**Figure 2:**
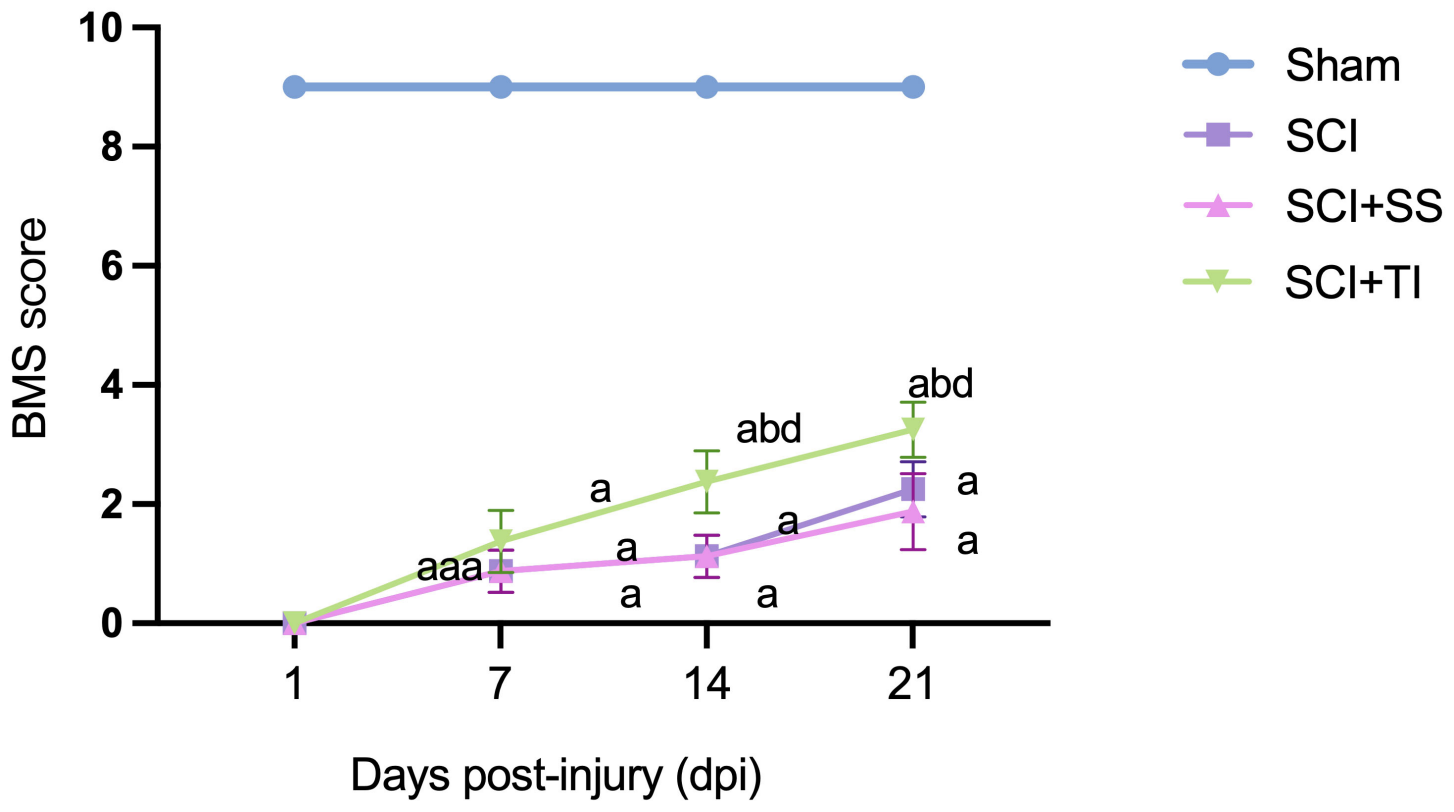
BMS scores of mice in each group over time. Data are presented as mean ± SD (*n* = 8 per group). Compared with the sham group, ^a^*P* < 0.05; compared with the SCI group, ^b^*P* < 0.05; compared with the SCI+SS group, ^d^*P* < 0.05.

### TI stimulation attenuates neuronal loss-associated changes after SCI

At 21 dpi, GFAP and NeuN immunofluorescence analysis was performed to assess astrocyte activation and neuronal loss-associated changes (Figure 3A). SCI induction resulted in a significant expansion of the GFAP-positive area in the SCI and SCI+SS groups relative to sham controls (Figure 3B) and a marked reduction in NeuN-positive neuronal density (both *P* < 0.05) (Figure 3C), indicating enhanced astroglial activation and reduced NeuN-positive neuronal density following SCI.

**Figure 3:**
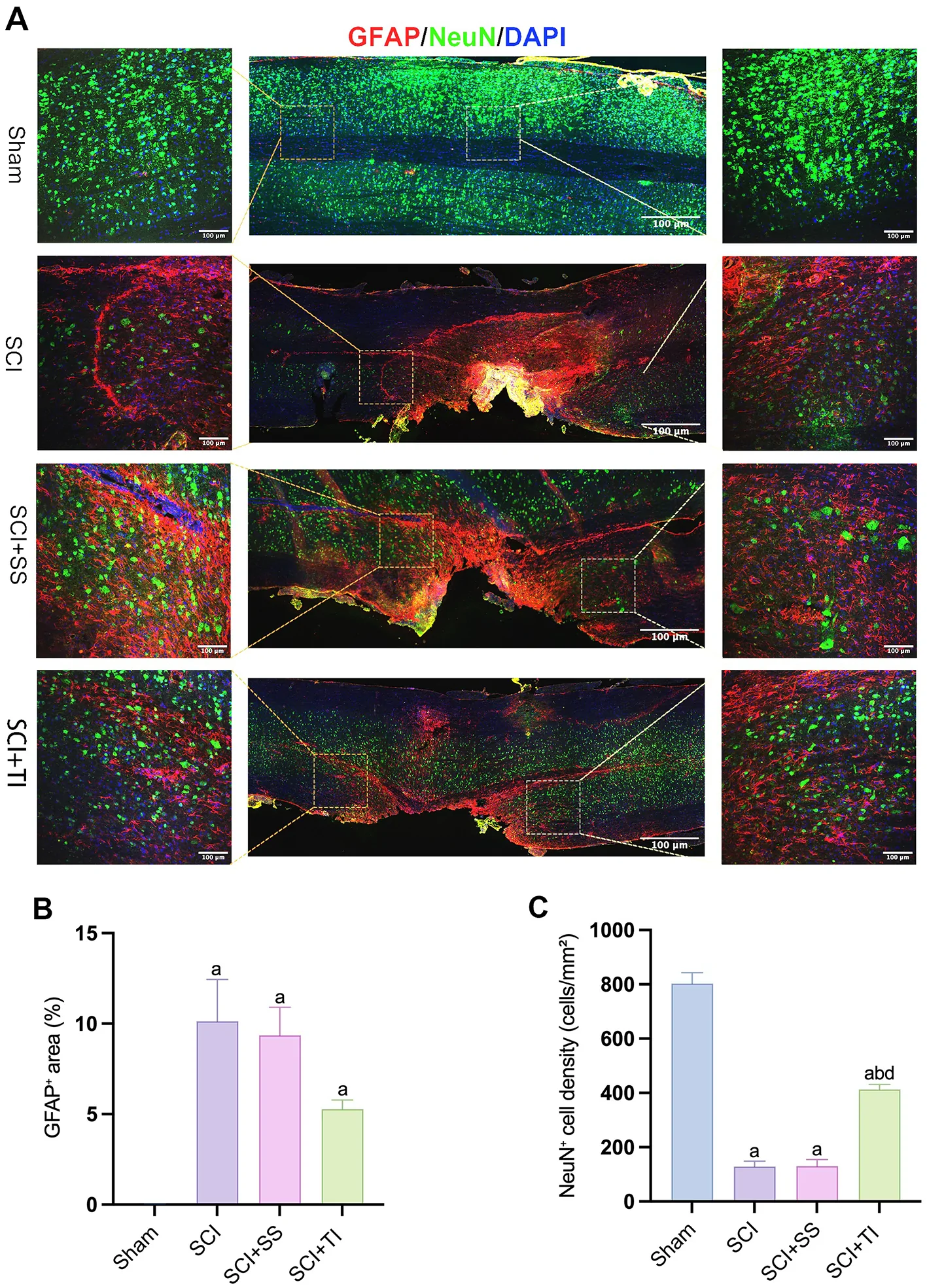
Immunofluorescence staining of GFAP and NeuN in the spinal cord of mice in each group. **(A)** Representative immunofluorescence images of GFAP and NeuN staining in spinal cord sections at 21 dpi. **(B)** Quantification of GFAP-positive area. **(C)** Quantification of NeuN-positive cell density. Data are presented as mean ± SD (*n* = 3 per group). Compared with the sham group, ^a^*P* < 0.05; compared with the SCI group, ^b^*P* < 0.05; compared with the SCI+SS group, ^d^*P* < 0.05.

Further analysis revealed that TI stimulation significantly increased NeuN-positive neuronal density in the SCI+TI group compared with both the SCI and SCI+SS groups (*P* < 0.05; Figure 3C). Meanwhile, GFAP-positive areas exhibited a decreasing tendency following TI stimulation, although the reduction did not reach statistical significance relative to the SCI and SCI+SS groups (*P* > 0.05; Figure 3B). These findings suggest that TI stimulation may attenuate neuronal loss-associated changes after SCI.

### TI stimulation enhances synaptic plasticity-associated protein expression after SCI

PSD95 and SYN immunofluorescence analysis was conducted at 21 dpi to assess synaptic plasticity-associated changes following SCI and TI stimulation (Figure 4A). Compared with sham controls, SCI-induced injury resulted in decreased PSD95 and SYN fluorescence intensity, as observed in both the SCI and SCI+SS conditions (both *P* < 0.05; Figures 4B, C).

**Figure 4:**
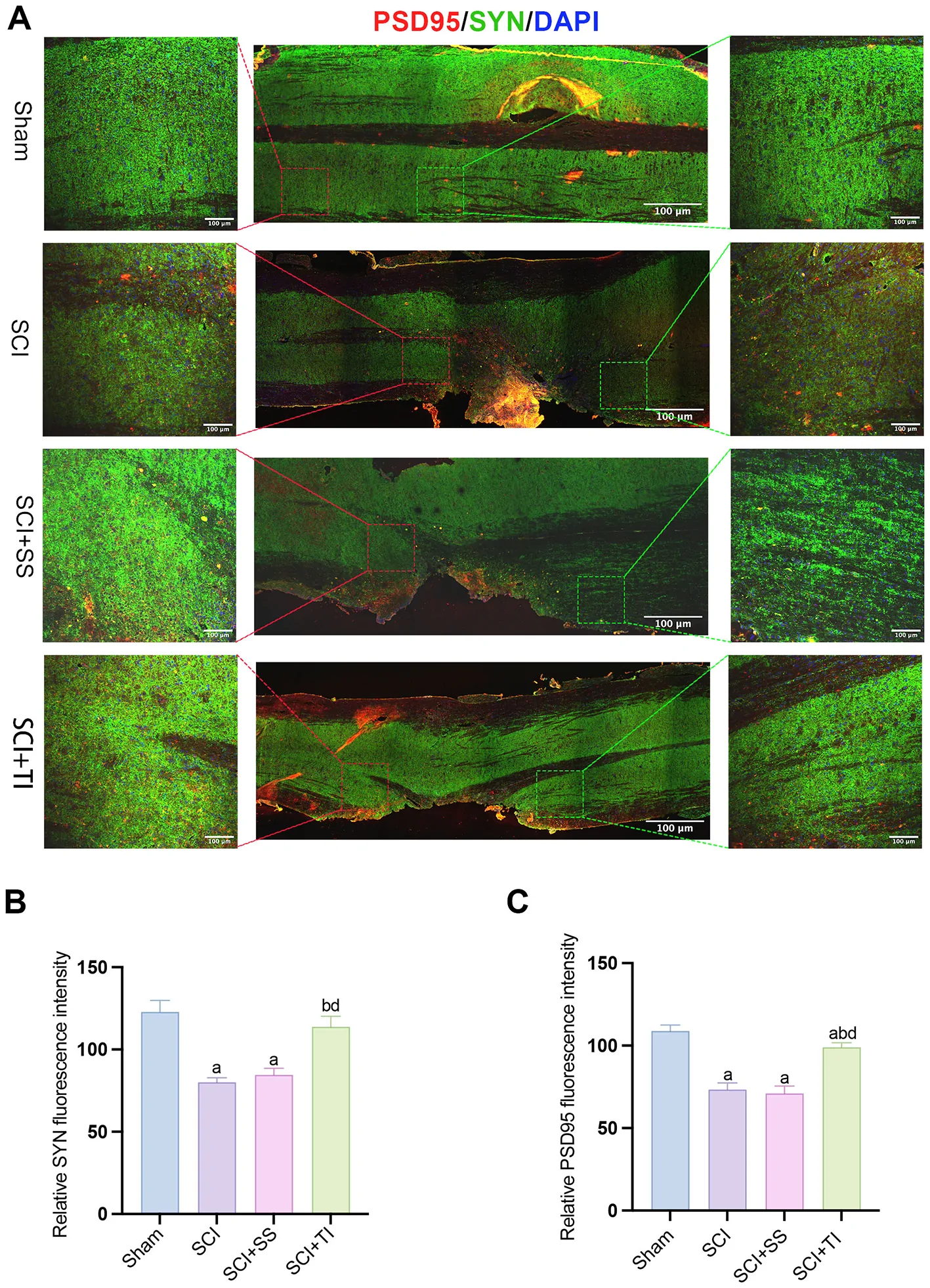
Immunofluorescence staining of PSD95 and SYN in spinal cord from each group. **(A)** Representative immunofluorescence images of PSD95 and SYN staining in spinal cord sections at 21 dpi. **(B)** Quantification of the mean fluorescence intensity of PSD95. **(C)** Quantification of the mean fluorescence intensity of SYN. Data are presented as mean ± SD (*n* = 3 per group). Compared with the sham group, ^a^*P* < 0.05; compared with the SCI group, ^b^*P* < 0.05; compared with the SCI+SS group, ^d^*P* < 0.05.

Following TI stimulation, the mean fluorescence intensities of PSD95 and SYN were markedly increased in the SCI+TI group compared with the SCI and SCI+SS groups (both *P* < 0.05; Figures 4B, C). These findings indicate that TI stimulation enhances synaptic plasticity-associated protein expression following SCI.

### TI stimulation increases the expression of axonal growth-associated and synaptic plasticity-related proteins after SCI

To characterize TI-mediated neural repair processes, axonal growth-associated markers and synaptic plasticity-related proteins were analyzed following SCI. The expression levels of GAP43, NF-L, PSD95, and SYN were assayed by Western blot at 21 dpi. Analysis revealed reduced protein expression of GAP43, NF-L, PSD95, and SYN in the SCI and SCI+SS groups relative to sham controls (all *P* < 0.05; Figure 5).

**Figure 5:**
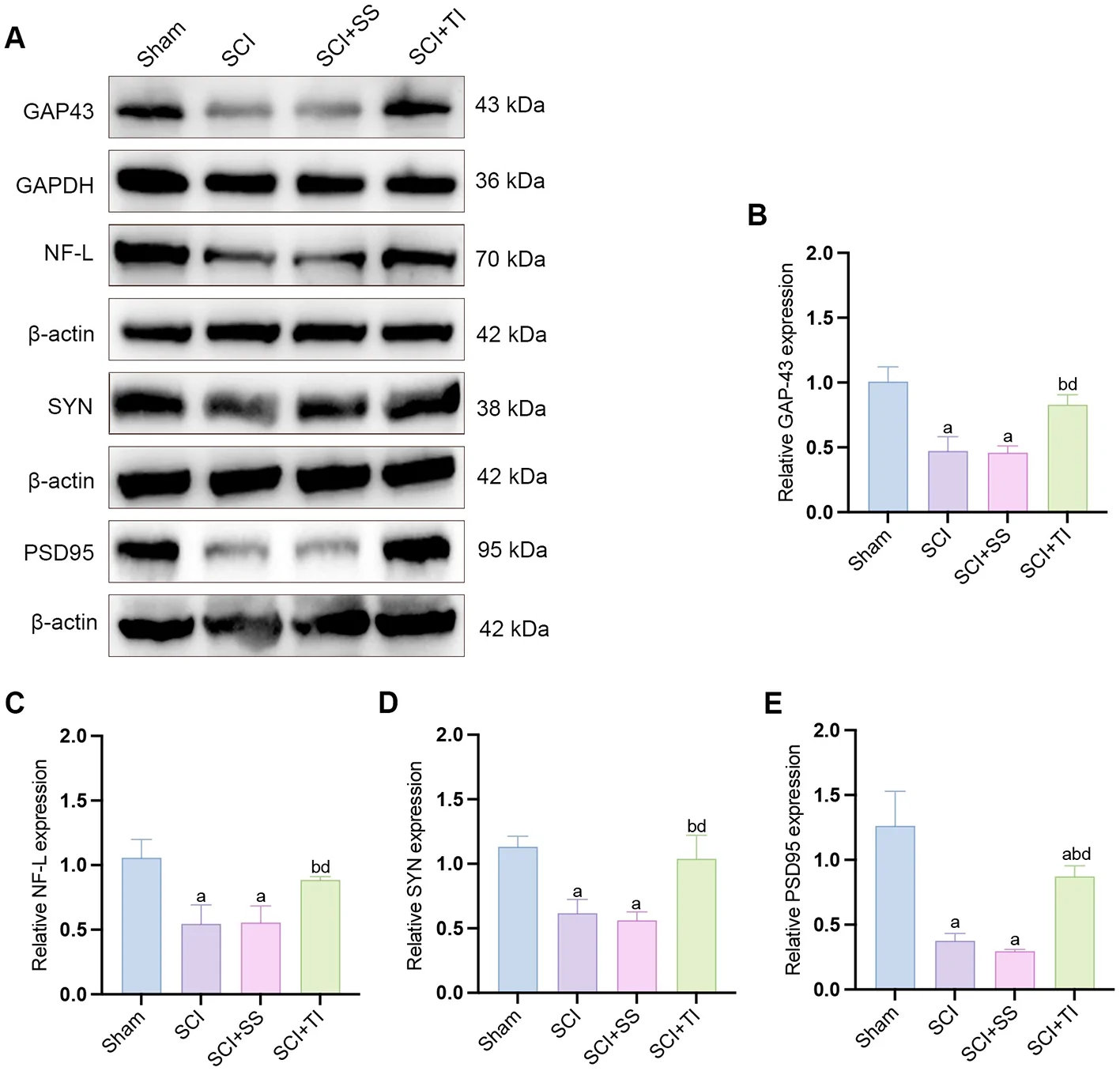
Western blot analysis of axonal growth-associated and synaptic plasticity-related proteins in spinal cord tissue from each group. **(A)** Representative Western blot images of GAP43, NF-L, PSD95, and SYN in spinal cord tissue at 21 dpi. GAPDH was used as the loading control for GAP43, whereas β-actin was used for NF-L, PSD95, and SYN. **(B–E)** Quantification of the relative protein expression levels of GAP43, NF-L, PSD95, and SYN. Data are presented as mean ± SD (*n* = 3 per group). Compared with the sham group, ^a^*P* < 0.05; compared with the SCI group, ^b^*P* < 0.05; compared with the SCI+SS group, ^d^*P* < 0.05.

Following TI stimulation, the expression levels of GAP43, NF-L, PSD95, and SYN were restored in the SCI+TI group compared with the untreated injury groups (all *P* < 0.05; Figure 5). These findings suggest that TI stimulation enhances axonal growth-associated and synaptic plasticity-related responses after SCI.

## Discussion

Spinal cord stimulation has been demonstrated to modulate spinal excitability and promote the functional reorganization of residual neural circuits after SCI, contributing to improved functional recovery (Minassian et al., 2016; Greiner et al., 2021). Sagalajev et al. (Sagalajev et al., 2024) found that EES not only increased the number of activated axons but also influenced neural signal transmission and network function by modulating the temporal patterns of axonal firing, suggesting that electrical stimulation regulates not only neuronal recruitment but also activity-dependent information processing within neural networks. Furthermore, studies have found that TSCS can modulate the spinal neural circuit excitability and supraspinal neural networks (Tajali et al., 2024). Minassian et al. (Minassian et al., 2016) found that TSCS transiently enhanced the function of spinal presynaptic and postsynaptic inhibitory circuits through the activation of Ia afferent fibers via transsynaptic mechanisms, further indicating that electrical stimulation can rapidly modulate the dysfunctional state of spinal circuits after injury. Beyond the modulation of neural activity, certain forms of electrical stimulation have also been reported to promote neural plasticity-associated processes and structural adaptations after injury (Jack et al., 2020; Choi et al., 2021; Samejima et al., 2022; Ali and Schwalb, 2024; Rowald et al., 2022; Sachdeva et al., 2026). EES has been reported to enhance synaptic plasticity and facilitate the recovery of residual neural circuits (Politis and Zanakis, 1988; Angelin et al., 2024), whereas oscillating field stimulation (OFS) promotes axonal protection and regeneration by providing directional electrical cues through periodic changes in electric field polarity (Borgens et al., 1999; Bacova et al., 2022; Wang et al., 2022).

We explored the effects of TI stimulation on functional recovery and n neuroplasticity-associated changes after SCI. Our findings showed that TI stimulation significantly improved hindlimb locomotor recovery in SCI mice, accompanied by increased NeuN-positive neuronal density and enhanced expression of axonal growth-associated and synaptic plasticity-related proteins in the injured spinal cord. Specifically, TI stimulation increased NeuN-positive neuronal density and upregulated the expression levels of GAP43, NF-L, SYN, and PSD95, which are closely associated with axonal growth, axonal structural integrity, and synaptic function. These findings demonstrate that TI stimulation improves functional recovery after SCI and is associated with enhanced neuroplasticity-related responses, including axonal growth-associated and synaptic plasticity-associated changes.

TI stimulation provides a novel means of achieving non-surgical modulation of neural function. Studies have found that TI stimulation improves the targeting selectivity of deep brain modulation, regulates the excitability of deep brain regions and neural network activity, and influences neural oscillatory patterns, demonstrating its potential for modulating neural function (Grossman et al., 2017; Esmaeilpour et al., 2021; Thiele et al., 2024; Vieira et al., 2024; Li et al., 2025; Wang et al., 2025a; Lamoš et al., 2025). Integrating these observations, it is evident that TI stimulation can alter neuronal activity states, while dynamic regulation of neural activity is considered an important basis for activity-dependent neuroplasticity (Martin et al., 2007; Pan-Vazquez et al., 2020). Further studies have found that TI stimulation with a 20 Hz envelope frequency (20 min per day for 7 consecutive days) improved motor skill performance in mice and was accompanied by synaptic plasticity-related changes, including molecular alterations associated with synaptic plasticity, structural remodeling of dendritic compartments, and enhanced presynaptic organization (Qi et al., 2024). Recent studies have explored the application of TI stimulation in spinal cord rehabilitation. Cheng et al. (Cheng et al., 2025) reported functional and neurophysiological improvements following TI stimulation in patients with incomplete cervical SCI using 1,000- and 1,040-Hz carrier frequencies, whereas Xie et al. (Xie et al., 2025) described a simulation-guided protocol for optimizing electrode placement for targeted transspinal TI stimulation. In contrast, the present study employed a mouse T8 spinal cord hemisection model with 2,000- and 2,020-Hz carrier frequencies and further evaluated behavioral, histological, and molecular changes. These findings complement previous clinical and methodological studies by providing additional evidence of neuroplasticity-associated responses following TI stimulation after SCI.

Consistent with the concept that TI-induced modulation of neural activity may influence activity-dependent plasticity, our study found that TI stimulation significantly increased the expression of the axonal growth-associated marker GAP43 and the axonal structural protein NF-L, while also elevating the levels of the synaptic-associated proteins PSD95 and SYN. These molecular changes suggest that TI stimulation may enhance axonal growth-associated responses and synaptic plasticity-related processes after SCI.

Despite these promising findings, several limitations should be acknowledged. First, although TI stimulation was associated with increased expression of neuroplasticity-related markers, including GAP43, NF-L, PSD95, and SYN, these molecular changes should be interpreted as indicators of axonal growth-associated and synaptic plasticity-related responses rather than direct evidence of axonal regeneration or synaptic reconstruction. The present study did not provide direct evidence of axonal regrowth, synaptic structural remodeling, or the specific neural mechanisms underlying functional recovery. Future studies incorporating axonal tracing, ultrastructural analysis, electrophysiological assessment, and circuit-level analyses are needed to further determine whether TI stimulation promotes structural reorganization and functional integration of spinal neural circuits. Second, although the sham stimulation group controlled for potential confounding effects associated with anesthesia, electrode placement, and experimental handling, the present study did not include an active stimulation control group receiving conventional electrical stimulation. Therefore, the current findings demonstrate the beneficial effects associated with TI stimulation but cannot completely exclude the possibility that some effects may be shared with other forms of electrical stimulation. Future studies with appropriate active stimulation controls are required to further clarify the specific contribution of the temporal interference mechanism to SCI recovery. Third, the molecular basis through which TI stimulation exerts its therapeutic effects has not been fully elucidated. Further investigations combining transcriptomic analysis, molecular validation, and pathway-specific interventions are required to identify the key cellular and signaling mechanisms involved in TI-mediated neural repair. In addition, the current study focused on a relatively early recovery phase after SCI, and longer-term studies are needed to evaluate the sustained effects and translational potential of TI stimulation. Future studies may also benefit from integrating advanced computational approaches to improve the precision and personalization of TI-based neuromodulation. Recent advances in artificial intelligence have demonstrated potential applications in anatomical analysis and diagnostic imaging, providing new opportunities for more precise characterization and modeling of biological structures (Choudhary et al., 2025; Choudhary, 2026). Integration of such approaches with computational modeling may, in the future, facilitate individualized stimulation planning and optimization of TI-based neuromodulation strategies.

In conclusion, we found that TI stimulation improves functional recovery after incomplete SCI, accompanied by increased NeuN-positive neuronal density and enhanced expression of axonal growth-associated and synaptic plasticity-related proteins.

## Data Availability

The raw data supporting the conclusions of this article will be made available by the authors, without undue reservation.
