# Supplementary material for: Transspinal temporal interference stimulation enhances neuroplasticity-associated responses and facilitates motor recovery after spinal cord injury in mice
Source: Front Neurosci. 2026 Oct 6;20:1950023. doi: 10.3389/fnins.2026.1950023 (PMC13680409; doi:10.3389/fnins.2026.1950023)
Supplement: Supplementary file 1 [file Data_Sheet_1.docx]

**Supplementary Figure**

**Supplementary Figure 1**

**Figure S1. Finite element modeling of transspinal temporal interference stimulation in the mouse spinal cord.**

**(A) Schematic illustration of the electrode configuration used for transspinal temporal interference stimulation. Two pairs of needle electrodes were symmetrically positioned along the dorsal surface of the spinal cord to generate two interfering electric fields targeting the T8 spinal cord region.**

**(B) Three-dimensional finite element model of the mouse body used for computational simulation. The model incorporated anatomical structures and electrode positions to calculate the spatial distribution of the induced electric fields during TI stimulation.**

**(C) Representative axial, sagittal, coronal, and three-dimensional dorsal views showing the simulated temporal interference electric field distribution in the mouse spinal cord. The color scale represents the maximum temporal interference electric field intensity, illustrating the spatial localization and penetration of the stimulation field around the T8 spinal cord segment.**
